# 335. *Staphylococcus aureus* Bacteremia as a Potential and Severe Complication from Intramuscular COVID-19 Vaccine Injection

**DOI:** 10.1093/ofid/ofab466.536

**Published:** 2021-12-04

**Authors:** Miguel Sebastian Pedromingo Kus

**Affiliations:** HOSPITAL NUESTRA SEÑORA DE SONSOLES, MADRID, Castilla y Leon, Spain

## Abstract

**Background:**

Abscess formation and bacteremia following intramuscular injections are rare complications from vaccine injections, and they are most commonly seen in immunocompromised individuals. *Staphylococcus aureus* is one of the etiological agents that can be found during this complication. Spain started to vaccine its population at the beginning of 2021. We noticed an important increase in *Staphylococcus aureus* infections and bacteremia during this period of time, leading us to study the relationship with previous vaccination.

**Methods:**

In this case series we present a cohort of twenty patients with *Staphylococcus aureus* bacteremia (SAB) during the study period (January 1, 2021 through May 31, 2021), attended in our Institution (Hospital Nuestra Señora de Sonsoles, Ávila, Spain). We tried to establish or at least create the debate of a possible relationship with a previous COVID-19 vaccine.

**Results:**

From January 1, 2021 through May 31, 2021, 20 SAB were identified in our Institution. 13/20 patients were vaccinated (all of them with the mRNA vaccine type). 5/13 (38%) were male and 8/13 (62%) female. 10 of them (77%) received at least one dose of the vaccine before hospital admission, and 3 of them (23%) after admission. From the 10 previously COVID-19-vaccinated patients treated for SAB (CVPSAB), 4 died - 40% (2 deaths directly related to the SAB).

**Conclusion:**

Although SAB may be a rare side effect after intramuscular injections or vaccines, it always implies an outstanding risk due to potential complications. Even if our study is not able to directly establish a link between SAB and previous vaccination, it implies a possible association between the vaccine injection and a threating disease (SAB). We should be aware of this probable relationship, so that we can maximize preventive measures.

**Disclosures:**

**All Authors**: No reported disclosures

